# Methicillin-resistant *Staphylococcus aureus*, Pakistan, 1996–2003

**DOI:** 10.3201/eid1009.030844

**Published:** 2004-09

**Authors:** Tariq Butt, Rifat Nadeem Ahmad, Muhammad Usman, Abid Mahmood

**Affiliations:** * Armed Forces Institute of Pathology, Rawalpindi, Pakistan

**Keywords:** letter, methicillin-resistant, *Staphylococcus aureus*, Pakistan

**To the Editor:** This letter is written in response to the article titled "Co-trimoxazole-sensitive, methicillin-resistant *Staphylococcus aureus*, Israel, 1988–1997" (*1*). We found the authors' findings most interesting. As the authors pointed out, methicillin-resistant *Staphylococcus aureus* (MRSA) infections have become a major problem worldwide. The problem is not restricted to industrialized countries. The last decade has seen an alarming increase in MRSA infections in Pakistani hospitals (*2*). Pakistan's Armed Forces Institute of Pathology provides laboratory services to a 1,500-bed tertiary-care hospital in Rawalpindi and is the main reference laboratory in northern Pakistan. According to our computerized database, the frequency of MRSA among all nosocomial isolates of *S. aureus* increased from 39% (212/543) in 1996 to 51% (516/1,018) in 2003 (p < 0.0001). Most of the isolates were obtained from pus and pus swab specimens (153 in 1996 and 394 in 2003), while the rest were obtained from blood (20 in 1996 and 37 in 2003), intravenous catheter tips and surgical drainage tubes (14 in 1996 and 31 in 2003), various body fluids (9 in 1996 and 19 in 2003), respiratory secretions (8 in 1996 and 18 in 2003), tissue (4 in 1996, 9 in 2003), throat swabs (2 in 1996, 6 in 2003), and urine (2 in 1996, 5 in 2003).

During the last 7 years, resistance in MRSA isolates has steadily increased to most of the antimicrobial drugs such as gentamicin (69% in 1996 and 88% in 2003), ciprofloxacin (87% in 1996 and 94% in 2003), clindamycin (60% in 1996 and 70% in 2003), and rifampicin (20% in 1996 and 60% in 2003). However, resistance to co-trimoxazole and doxycycline has decreased. In 1996, 15% (32/212) of our MRSA isolates were susceptible to co-trimoxazole, whereas in the first 9 months of 2003, 43% (222/516) of the isolates were susceptible (p < 0.0001). Similarly, susceptibility to doxycycline increased from 34% in 1996 to 49% in 2003 (p = 0.0005). Antimicrobial drug susceptibility of the isolates was tested by the modified Kirby-Bauer technique and results were interpreted according to the National Committee for Clinical Laboratory Standards criteria (*3*). Methicillin resistance was tested by using 1 µg oxacillin disks (Oxoid, Basingstoke, Hampshire, UK) on Mueller-Hinton agar containing 4% sodium chloride. Plates were incubated at 35°C for 24 hours.

We agree with Bishara et al. (*1*) that the increase in susceptibility is likely due to decreased use of these antimicrobial drugs for staphylococcal infections in clinical practice. The use of co-trimoxazole in our hospital decreased from 48 daily doses per 1,000 hospital days in 1996 to 35 daily doses in 2003, while use of doxycycline decreased from 12 daily doses per 1,000 hospital days in 1996 to 9 daily doses in 2003 (*4*). These antimicrobial drugs offer an inexpensive alternative to glycopeptides for the treatment of MRSA infections. Data from the United States and Europe have shown that vancomycin-intermediate *S. aureus* isolates also remain susceptible to some of the conventional antimicrobial drugs, including co-trimoxazole (*5*). If their efficacy in vivo is validated by clinical trials, use of these conventional drugs would not only reduce the load on overstretched health care budgets but reduce the use of vancomycin, therefore decreasing the risk of isolates continuing to develop vancomycin resistance.
